# Fibromyalgia: functional, autoimmune or both? Treatment options for drug-resistant fibromyalgia

**DOI:** 10.1192/j.eurpsy.2024.1349

**Published:** 2024-08-27

**Authors:** C. P. Desport, D. O. Martins, J. R. Freitas, L. C. de Castro

**Affiliations:** Psychiatry, Hospital de Magalhães Lemos, Centro Hospitalar e Universitário do Porto, Porto, Portugal

## Abstract

**Introduction:**

fibromyalgia is a modern disease, with growing investigation concerning its etiology and treatment. It has become a very prevalent diagnosis and total remission of symptoms is the exception which is dramatic considering the socio-occupational impact of this highly debilitating disease.

**Objectives:**

to review the updates in the pathophysiology and treatment of fibromyalgia, especially when it is refractory to treatment. The authors also intend to better understand where fibromyalgia belongs, is it in psychiatry as a functional disorder or in rheumatology as an auto-immune disease?

**Methods:**

bibliographical search in PubMed database, using the key-words “fibromyalgia” and “psychiatry”, limited to works published in the last 10 years.

**Results:**

from our search resulted 158 articles, from reading of abstracts 30 were chosen for further reading.

**Conclusions:**

concerning the etiology of this disease, on the one hand psychological factors cannot be neglected since there are several studies finding a positive correlation between stressors like history of physical abuse and fibromyalgia in adulthood, on the other hand investigation and meta-analysis have found that the immune-inflammatory response system might be altered with dysregulation of pro and anti-inflammatory cytokines and cell-mediated immunity. Regarding treatment, symptom relief is often unsatisfactory with classical treatment and so adjunct treatment such as electrical neuromodulation and aerobic exercise might, respectively, be effective in reducing pain and depressive symptoms, thereby improving quality of life, and in improving fatigue and in a lesser degree sleep.

**Disclosure of Interest:**

None Declared

